# 2-[(Di­methyl­phenyl­phosphanyl­idene)aza­nium­yl]-5-methyl­benzene­sulfonate benzene monosolvate

**DOI:** 10.1107/S1600536813013755

**Published:** 2013-05-25

**Authors:** Christopher T. Burns, Suisheng Shang, Mark S. Mashuta

**Affiliations:** a Department of Chemistry, University of Louisville, Louisville, KY 40292, USA

## Abstract

The title compound, C_15_H_18_NO_3_PS·C_6_H_6_, is a rare example of a crystallographically characterized exocyclic phosphiniminium–arene­sulfonate zwitterion, which crystallises as its benzene solvate. The crystal structure shows that the N atom is protonated and that the iminium H atom forms both intra- and inter­molecular hydrogen bonds to the single-bonded sulfonate O atom in an *R*_2_^2^(4) graph-set motif. The dihedral angle between the aromatic rings in the main molecule is 89.49 (8)°.

## Related literature

For background to this class of compound, see: Brown *et al.* (2007 ▶); Bruneau & Achard (2012 ▶); Drent *et al.* (2002 ▶); Lee & Hoveyda (2009 ▶); Lee *et al.* (2008 ▶, 2009 ▶); Nakamura *et al.* (2009 ▶). For related structures, see: Burns *et al.* (2012 ▶); Liu *et al.* (1995 ▶); Perrotin *et al.* (2011 ▶); Spencer *et al.* (2003 ▶); Wallis *et al.* (2009 ▶, 2010 ▶); Zhang *et al.* (2006 ▶); Zhou & Jordan (2011 ▶). For hydrogen-bonding details, see: Desiraju (1995 ▶). 
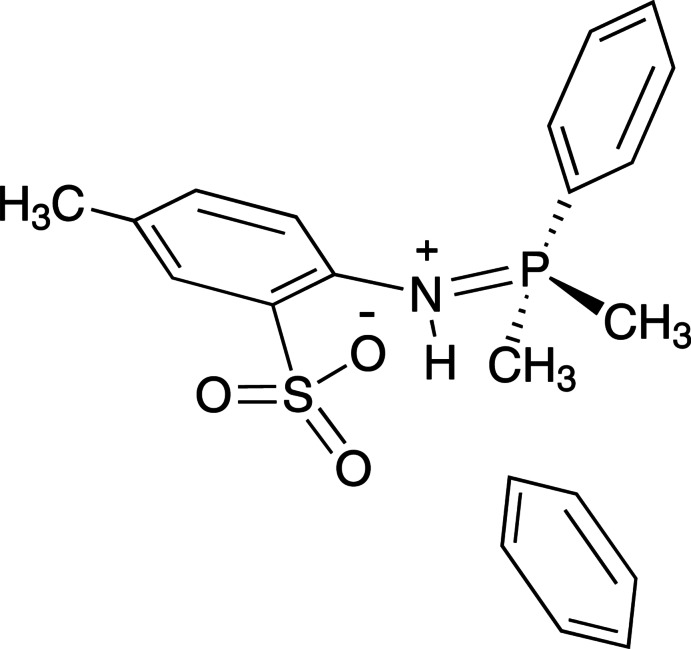


## Experimental

### 

#### Crystal data

C_15_H_18_NO_3_PS·C_6_H_6_
*M* *_r_* = 401.44
Triclinic, 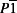

*a* = 9.3696 (2) Å
*b* = 10.3141 (2) Å
*c* = 11.7579 (3) Å
α = 68.665 (2)°
β = 78.180 (2)°
γ = 70.630 (2)°
*V* = 993.89 (4) Å^3^
*Z* = 2
Mo *K*α radiation
μ = 0.27 mm^−1^
*T* = 100 K
0.41 × 0.31 × 0.25 mm

#### Data collection

Agilent Xcalibur (Ruby, Gemini) diffractometer
Absorption correction: multi-scan (*CrysAlis PRO*; Agilent, 2010 ▶) *T*_min_ = 0.939, *T*_max_ = 1.000
22626 measured reflections
5263 independent reflections
4918 reflections with *I* > 2σ(*I*)
*R*_int_ = 0.017

#### Refinement

*R*[*F* ^2^ > 2σ(*F* ^2^)] = 0.041
*wR*(*F* ^2^) = 0.096
*S* = 1.05
5263 reflections
251 parameters
H atoms treated by a mixture of independent and constrained refinement
Δρ_max_ = 1.40 e Å^−3^
Δρ_min_ = −0.42 e Å^−3^

### 

Data collection: *CrysAlis PRO* (Agilent, 2010 ▶); cell refinement: *CrysAlis PRO*; data reduction: *CrysAlis PRO*; program(s) used to solve structure: *SHELXS97* (Sheldrick, 2008 ▶); program(s) used to refine structure: *SHELXL97* (Sheldrick, 2008 ▶); molecular graphics: *ORTEP-3 for Windows* (Farrugia, 2012 ▶) and *Mercury* (Macrae *et al.*, 2006 ▶); software used to prepare material for publication: *PLATON* (Spek, 2009 ▶) and *publCIF* (Westrip, 2010 ▶).

## Supplementary Material

Crystal structure: contains datablock(s) global, I. DOI: 10.1107/S1600536813013755/pk2483sup1.cif

Structure factors: contains datablock(s) I. DOI: 10.1107/S1600536813013755/pk2483Isup2.hkl

Supplementary material file. DOI: 10.1107/S1600536813013755/pk2483Isup3.cml

Additional supplementary materials:  crystallographic information; 3D view; checkCIF report

## Figures and Tables

**Table 1:** Hydrogen-bond geometry (Å, °)

*D*—H⋯*A*	*D*—H	H⋯*A*	*D*⋯*A*	*D*—H⋯*A*
N1—H1*N*⋯O2	0.80 (2)	2.09 (2)	2.7374 (17)	139 (2)
N1—H1*N*⋯O2^i^	0.80 (2)	2.47 (2)	3.0311 (17)	128 (2)

Symmetry code: (i) 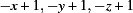.

**Fig. 1.**
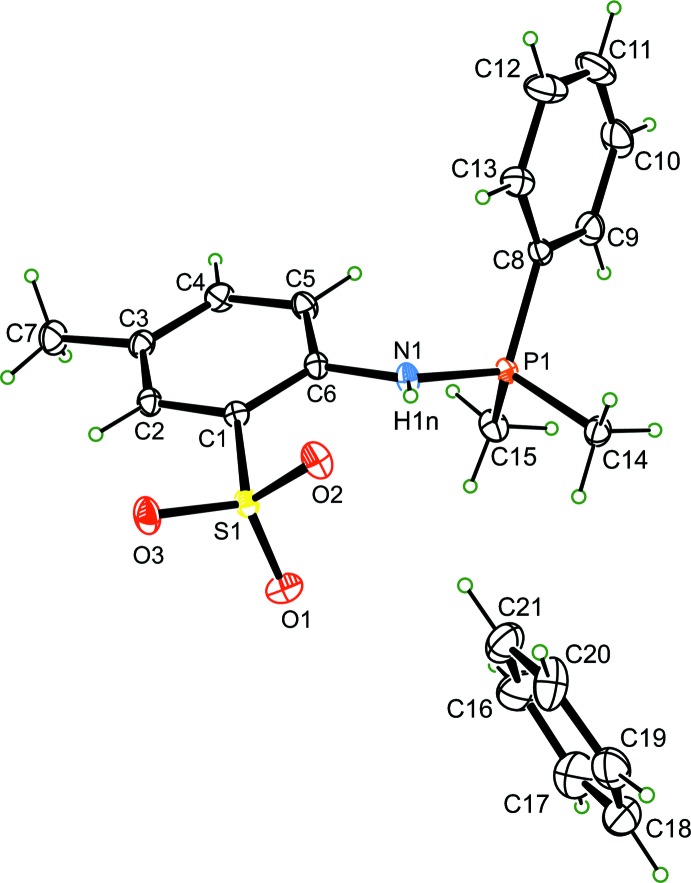
An ellipsoid plot of (I) showing 50% displacement ellipsoids. H atoms are shown as small spheres of arbitrary radii.

**Fig. 2.**
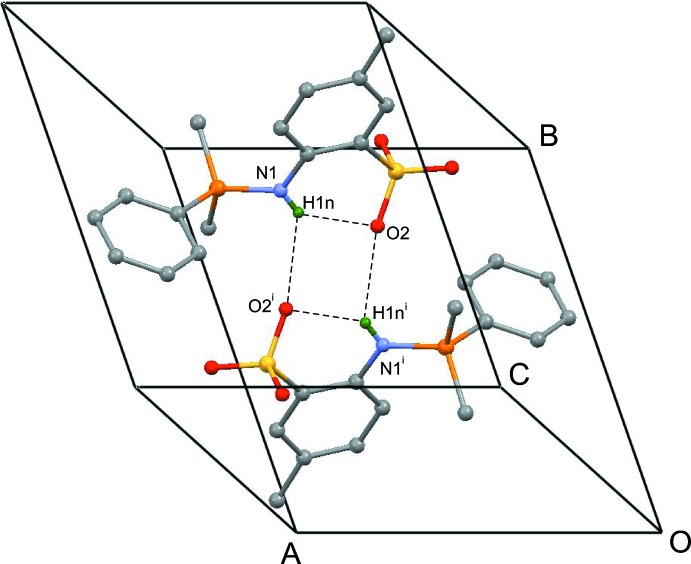
Packing diagram displaying intramolecular and intermolecular hydrogen-bonding interactions between H1n, O2 and O2i. [Symmetry code: (i) 1 - x, 1 - y, 1 - z]

## supplementary crystallographic information

### Comment

Exocyclic phosphinimine functionalized benzenesulfonate ligands have the potential to be used in the synthesis of new transition and main group metal catalysts with numerous applications. Few examples exist where an exocyclic phosphinimine has been incorporated into a mixed donor ligand system for use in the preparation of metal complexes (Liu *et al.*, 1995; Spencer *et al.*, 2003; Zhang *et al.*, 2006; Wallis *et al.*, 2009, 2010). There are also a very limited number of *ortho*-substituted arenesulfonate bidentate ligands used for catalytic reactions. 2-Phosphine-arenesulfonate chelates have received considerable attention in the last decade as ancillary ligands for group 10 olefin insertion polymerization catalysts (Drent *et al.*, 2002; Nakamura *et al.*, 2009; Perrotin *et al.*, 2011) and have also been used to stabilize ruthenium complexes that catalyze allylic alkylations of heterocycles and amines (Bruneau & Achard, 2012). Chiral and achiral *ortho*-*N*-heterocyclic-carbene-benzenesulfonate ligands have been used in the copper-catalyzed asymmetric conjugate addition, allylic alkylation, hydroboration and diboronation reactions (Brown *et al.*, 2007; Lee *et al.*, 2008, 2009; Lee & Hoveyda, 2009) as well as being explored as catalysts that promote insertion of unsaturated molecules into a palladium carbon bond (Zhou & Jordan, 2011). The title compound, (I), has been synthesized as an air-stable precursor of 2-dimethyl(phenyl)phosphinimine-5-methylbenzenesulfonate which is being explored as an ancillary ligand for metal mediated transformations.

The structures of the related 2-triphenylphosphiniminium-5-methylbenzenesulfonate and 2-diphenyl(methyl)phosphiniminium-5-methylbenzenesulfonate (Burns *et al.*, 2012), have been reported recently and contain the same primary structural features as those found in (I). All three structures confirm the zwitterionic nature of these compounds *via* presence of the phosphiniminium group in which the nitrogen is protonated and the sulfonate anion is located *ortho* to the protonated phosphinimine. The P(1)—N(1) bond distance (Table 1) in (I) is statistically the same as the phosphiniminium P—N bonds in both 2-triphenylphosphiniminium-5-methylbenzenesulfonate [1.6327 (17) Å] and 2-diphenyl(methyl)phosphiniminium-5-methylbenzenesulfonate [1.6380 (12) Å] (Burns *et al.*, 2012) indicative of single bond character. The iminium hydrogen forms a strong intramolecular hydrogen bond to one sulfonate oxygen atom N1—H1n- - –O2 (Table 2), (Desiraju, 1995). This hydrogen is also involved in an intermolecular interaction with the nearest symmetry generated O2 atom N1—H1n- - –O2^i^ (Table 2). The smaller phosphiniminium Me_2_PPh substituent in (I) has much less steric impact than the Ph_3_P and Ph_2_PMe groups in the two previously reported zwitterions. Comparison of relevant torsion angles in the three structures shows that the phosphorous atom of the phosphiniminium group in (I) is situated above the plane of the aryl ring of the 5-methylbenzenesulfonate fragment with a C5—C6—N1—P1 torsion angle of 29.7 (2)° whereas the phosphorous atom of the phosphiniminium group in the triphenylphosphiniminium and diphenyl(methyl)phosphiniminium zwitterion structures is located below the plane of the aryl ring with corresponding torsion angles of 39.2 (3)° and 14.9 (2)° respectively (Burns *et al.*, 2012).

### Experimental

Compound (I) was synthesized following a previously reported procedure (Burns *et al.*, 2012). Dimethylphenylphosphine (11.1 mmol) was added dropwise to a toluene solution (40 ml) of propyl-2-azido-5-methylbenzenesulfonate (9.3 mmol) at 298 K. Effervescence was observed immediately after addition of the phosphine and the clear yellow solution was stirred under N_2_. After 2 h the solvent was removed under vacuum. The resulting yellow oil was dissolved in CH_2_Cl_2_ (40 ml) and transferred *via* cannula to a suspension of pyridinium tetrafluoroborate (9.3 mmol) in CH_2_Cl_2_ (40 ml). Pyridine (14.0 mmol) was added to the white suspension in CH_2_Cl_2_ and the reaction mixture was stirred at 298 K for 48 h. A clear pale green solution was observed and the volatiles were removed under vacuum. The resulting residue was treated with diethyl ether (4 *x* 60 ml) followed by filtration to give a light yellow solid. The solid was purified *via* column chromatography using silica gel (160 g) and CH_2_Cl_2_. The column was eluted with CH_2_Cl_2_:MeOH 95:5 (500 ml), CH_2_Cl_2_:MeOH 90:10 (1 *L*) and CH_2_Cl_2_:MeOH 80:20 (1 *L*). The fractions (100 ml) were analyzed by UV-Vis, and those that contained the product were combined and dried under vacuum, affording a white solid. The solid was treated with CH_2_Cl_2_ (20 ml) and diethyl ether (100 ml) to afford a white precipitate. Filtration of the precipitate followed by diethyl ether washes (3 *x* 20 ml) afforded the title compound as a white powder (3.86 g, 70%). Crystals for X-ray analysis were obtained by slow diffusion of benzene into a CHCl_3_ solution of the title compound in a sealed 5 mm tube at 298 K.

### Refinement

The iminum hydrogen atom was located in a difference map and refined isotropically. Aromatic H atom positions were calculated, and included as fixed contributions with *U*_iso_(H) = 1.2 *x U*_eq_(C). Methyl H atoms were placed in calculated positions and allowed to ride (the torsion angle which defines its orientation was allowed to refine) on the attached C atom, and these atoms were assigned *U*_iso_(H) = 1.5 *x U*_eq_(C). The highest peak, 1.40 e/Å^3^, and deepest trough, -0.42 e/Å^3^, are located 0.75 Å and 0.73 Å from C16 and C20 respectively.

### Crystal data

**Table:**

C_15_H_18_NO_3_PS·C_6_H_6_	*Z* = 2
*M**_r_* = 401.44	*F*(000) = 424
Triclinic, *P*1	*D*_x_ = 1.341 Mg m^−^^3^
Hall symbol: -P 1	Melting point: 490 K
*a* = 9.3696 (2) Å	Mo *K*α radiation, λ = 0.71073 Å
*b* = 10.3141 (2) Å	Cell parameters from 16324 reflections
*c* = 11.7579 (3) Å	θ = 3.3–29.0°
α = 68.665 (2)°	µ = 0.27 mm^−^^1^
β = 78.180 (2)°	*T* = 100 K
γ = 70.630 (2)°	Prism, colorless
*V* = 993.89 (4) Å^3^	0.41 × 0.31 × 0.25 mm

### Data collection

**Table:**

Agilent Xcalibur (Ruby, Gemini) diffractometer	5263 independent reflections
Radiation source: Enhance (Mo) X-ray Source	4918 reflections with *I* > 2σ(*I*)
Graphite monochromator	*R*_int_ = 0.017
Detector resolution: 10.2836 pixels mm^-1^	θ_max_ = 29.1°, θ_min_ = 3.3°
ω scans	*h* = −12→12
Absorption correction: multi-scan (*CrysAlis PRO*; Agilent, 2010)	*k* = −13→13
*T*_min_ = 0.939, *T*_max_ = 1.000	*l* = −16→16
22626 measured reflections	

### Refinement

**Table:**

Refinement on *F*^2^	Primary atom site location: structure-invariant direct methods
Least-squares matrix: full	Secondary atom site location: difference Fourier map
*R*[*F*^2^ > 2σ(*F*^2^)] = 0.041	Hydrogen site location: inferred from neighbouring sites
*wR*(*F*^2^) = 0.096	H atoms treated by a mixture of independent and constrained refinement
*S* = 1.05	*w* = 1/[σ^2^(*F*_o_^2^) + (0.0264*P*)^2^ + 1.4298*P*] where *P* = (*F*_o_^2^ + 2*F*_c_^2^)/3
5263 reflections	(Δ/σ)_max_ = 0.001
251 parameters	Δρ_max_ = 1.40 e Å^−^^3^
0 restraints	Δρ_min_ = −0.42 e Å^−^^3^

### Fractional atomic coordinates and isotropic or equivalent isotropic displacement parameters (Å^2^)

**Table:**

	*x*	*y*	*z*	*U*_iso_*/*U*_eq_	
S1	0.24462 (4)	0.76594 (4)	0.45953 (3)	0.01262 (9)	
P1	0.71909 (4)	0.68470 (4)	0.55802 (3)	0.01128 (9)	
O1	0.28714 (14)	0.88299 (13)	0.35938 (11)	0.0211 (2)	
O2	0.34682 (13)	0.62271 (12)	0.46153 (11)	0.0181 (2)	
O3	0.08543 (12)	0.77151 (14)	0.47013 (11)	0.0200 (2)	
N1	0.54554 (14)	0.67514 (14)	0.56918 (12)	0.0135 (2)	
H1N	0.530 (3)	0.632 (3)	0.531 (2)	0.027 (6)*	
C1	0.27173 (16)	0.78953 (15)	0.59586 (13)	0.0119 (3)	
C2	0.14789 (16)	0.86274 (16)	0.65917 (14)	0.0139 (3)	
H2	0.0516	0.8904	0.6336	0.017*	
C3	0.16470 (17)	0.89556 (16)	0.75995 (14)	0.0155 (3)	
C4	0.30965 (18)	0.84905 (17)	0.79818 (14)	0.0171 (3)	
H4	0.3235	0.8684	0.8658	0.021*	
C5	0.43403 (17)	0.77421 (17)	0.73725 (14)	0.0156 (3)	
H5	0.5294	0.7428	0.7655	0.019*	
C6	0.41761 (16)	0.74565 (15)	0.63435 (13)	0.0125 (3)	
C7	0.03151 (19)	0.98321 (19)	0.82218 (16)	0.0221 (3)	
H7A	0.0137	0.9262	0.9060	0.033*	
H7B	−0.0573	1.0103	0.7806	0.033*	
H7C	0.0532	1.0690	0.8195	0.033*	
C8	0.81146 (16)	0.56744 (16)	0.69318 (13)	0.0140 (3)	
C9	0.92555 (18)	0.6000 (2)	0.72847 (15)	0.0210 (3)	
H9	0.9523	0.6850	0.6835	0.025*	
C10	0.9990 (2)	0.5043 (2)	0.83147 (17)	0.0296 (4)	
H10	1.0755	0.5250	0.8552	0.036*	
C11	0.9585 (2)	0.3787 (2)	0.89842 (18)	0.0329 (4)	
H11	1.0074	0.3155	0.9676	0.040*	
C12	0.8455 (2)	0.34585 (19)	0.86339 (17)	0.0286 (4)	
H12	0.8190	0.2609	0.9090	0.034*	
C13	0.77174 (19)	0.43981 (17)	0.76011 (15)	0.0191 (3)	
H13	0.6966	0.4177	0.7360	0.023*	
C14	0.82093 (17)	0.63095 (17)	0.42977 (14)	0.0169 (3)	
H14A	0.8261	0.5318	0.4443	0.025*	
H14B	0.9220	0.6403	0.4181	0.025*	
H14C	0.7698	0.6918	0.3577	0.025*	
C15	0.71858 (18)	0.86602 (16)	0.53121 (16)	0.0186 (3)	
H15A	0.6603	0.9301	0.4637	0.028*	
H15B	0.8210	0.8728	0.5121	0.028*	
H15C	0.6741	0.8931	0.6034	0.028*	
C16	0.6085 (3)	0.8347 (2)	0.14006 (19)	0.0392 (5)	
H16	0.5942	0.8883	0.1923	0.047*	
C17	0.6651 (3)	0.8839 (3)	0.0207 (2)	0.0363 (5)	
H17	0.6838	0.9741	−0.0092	0.044*	
C18	0.6951 (2)	0.8019 (2)	−0.05696 (18)	0.0318 (4)	
H18	0.7352	0.8361	−0.1377	0.038*	
C19	0.6647 (2)	0.6685 (2)	−0.01315 (18)	0.0296 (4)	
H19	0.6886	0.6115	−0.0639	0.035*	
C20	0.6011 (2)	0.6207 (2)	0.1021 (2)	0.0319 (4)	
H20	0.5761	0.5336	0.1287	0.038*	
C21	0.5720 (2)	0.7022 (2)	0.18328 (18)	0.0311 (4)	
H21	0.5297	0.6686	0.2634	0.037*	

### Atomic displacement parameters (Å^2^)

**Table:**

	*U*^11^	*U*^22^	*U*^33^	*U*^12^	*U*^13^	*U*^23^
S1	0.01064 (16)	0.01423 (17)	0.01497 (16)	−0.00342 (12)	−0.00207 (12)	−0.00656 (13)
P1	0.00971 (16)	0.01120 (16)	0.01365 (17)	−0.00251 (12)	−0.00188 (12)	−0.00481 (13)
O1	0.0269 (6)	0.0214 (6)	0.0159 (5)	−0.0104 (5)	−0.0009 (4)	−0.0045 (4)
O2	0.0163 (5)	0.0174 (5)	0.0248 (6)	−0.0016 (4)	−0.0054 (4)	−0.0125 (4)
O3	0.0117 (5)	0.0294 (6)	0.0234 (6)	−0.0056 (4)	−0.0028 (4)	−0.0131 (5)
N1	0.0098 (5)	0.0159 (6)	0.0188 (6)	−0.0028 (4)	−0.0019 (4)	−0.0107 (5)
C1	0.0122 (6)	0.0112 (6)	0.0137 (6)	−0.0043 (5)	−0.0013 (5)	−0.0045 (5)
C2	0.0118 (6)	0.0129 (6)	0.0169 (7)	−0.0039 (5)	−0.0005 (5)	−0.0047 (5)
C3	0.0155 (7)	0.0143 (6)	0.0158 (7)	−0.0035 (5)	0.0012 (5)	−0.0059 (5)
C4	0.0198 (7)	0.0183 (7)	0.0154 (7)	−0.0044 (6)	−0.0017 (6)	−0.0085 (6)
C5	0.0134 (6)	0.0173 (7)	0.0174 (7)	−0.0027 (5)	−0.0039 (5)	−0.0072 (6)
C6	0.0115 (6)	0.0107 (6)	0.0156 (6)	−0.0031 (5)	−0.0008 (5)	−0.0048 (5)
C7	0.0186 (7)	0.0250 (8)	0.0221 (8)	−0.0009 (6)	0.0012 (6)	−0.0130 (6)
C8	0.0119 (6)	0.0151 (7)	0.0144 (6)	−0.0008 (5)	−0.0021 (5)	−0.0065 (5)
C9	0.0152 (7)	0.0303 (9)	0.0206 (7)	−0.0075 (6)	−0.0026 (6)	−0.0102 (7)
C10	0.0191 (8)	0.0455 (11)	0.0260 (9)	−0.0008 (7)	−0.0100 (7)	−0.0166 (8)
C11	0.0346 (10)	0.0329 (10)	0.0227 (9)	0.0095 (8)	−0.0151 (7)	−0.0094 (7)
C12	0.0417 (11)	0.0169 (8)	0.0206 (8)	0.0001 (7)	−0.0079 (7)	−0.0033 (6)
C13	0.0234 (8)	0.0155 (7)	0.0184 (7)	−0.0037 (6)	−0.0037 (6)	−0.0061 (6)
C14	0.0146 (7)	0.0204 (7)	0.0159 (7)	−0.0039 (6)	0.0004 (5)	−0.0078 (6)
C15	0.0174 (7)	0.0121 (7)	0.0266 (8)	−0.0046 (5)	−0.0031 (6)	−0.0058 (6)
C16	0.0531 (13)	0.0367 (11)	0.0249 (9)	−0.0015 (10)	−0.0034 (9)	−0.0168 (8)
C17	0.0350 (11)	0.0372 (11)	0.0396 (11)	−0.0124 (9)	−0.0016 (9)	−0.0145 (9)
C18	0.0265 (9)	0.0423 (11)	0.0248 (9)	−0.0089 (8)	−0.0007 (7)	−0.0104 (8)
C19	0.0248 (9)	0.0381 (10)	0.0286 (9)	−0.0033 (8)	−0.0065 (7)	−0.0167 (8)
C20	0.0221 (9)	0.0338 (10)	0.0443 (11)	−0.0117 (7)	0.0074 (8)	−0.0199 (9)
C21	0.0268 (9)	0.0488 (12)	0.0215 (8)	−0.0143 (8)	0.0017 (7)	−0.0144 (8)

### Geometric parameters (Å, º)

**Table:**

S1—O1	1.4474 (12)	C9—H9	0.9300
S1—O3	1.4547 (11)	C10—C11	1.380 (3)
S1—O2	1.4648 (11)	C10—H10	0.9300
S1—O2	1.4648 (11)	C11—C12	1.387 (3)
S1—C1	1.7845 (15)	C11—H11	0.9300
P1—N1	1.6373 (13)	C12—C13	1.391 (2)
P1—C14	1.7776 (15)	C12—H12	0.9300
P1—C15	1.7783 (16)	C13—H13	0.9300
P1—C8	1.7911 (15)	C14—H14A	0.9600
N1—C6	1.4155 (18)	C14—H14B	0.9600
N1—H1N	0.80 (2)	C14—H14C	0.9600
C1—C2	1.395 (2)	C15—H15A	0.9600
C1—C6	1.4043 (19)	C15—H15B	0.9600
C2—C3	1.395 (2)	C15—H15C	0.9600
C2—H2	0.9300	C16—C17	1.365 (3)
C3—C4	1.392 (2)	C16—C21	1.406 (3)
C3—C7	1.506 (2)	C16—H16	0.9300
C4—C5	1.390 (2)	C17—C18	1.388 (3)
C4—H4	0.9300	C17—H17	0.9300
C5—C6	1.393 (2)	C18—C19	1.386 (3)
C5—H5	0.9300	C18—H18	0.9300
C7—H7A	0.9600	C19—C20	1.346 (3)
C7—H7B	0.9600	C19—H19	0.9300
C7—H7C	0.9600	C20—C21	1.421 (3)
C8—C13	1.394 (2)	C20—H20	0.9300
C8—C9	1.396 (2)	C21—H21	0.9300
C9—C10	1.391 (2)		
			
O1—S1—O3	114.14 (7)	C10—C9—C8	119.49 (17)
O1—S1—O2	112.97 (7)	C10—C9—H9	120.3
O3—S1—O2	112.35 (7)	C8—C9—H9	120.3
O1—S1—O2	112.97 (7)	C11—C10—C9	120.11 (17)
O3—S1—O2	112.35 (7)	C11—C10—H10	119.9
O1—S1—C1	105.48 (7)	C9—C10—H10	119.9
O3—S1—C1	106.09 (7)	C10—C11—C12	120.53 (17)
O2—S1—C1	104.84 (7)	C10—C11—H11	119.7
O2—S1—C1	104.84 (7)	C12—C11—H11	119.7
N1—P1—C14	107.43 (7)	C11—C12—C13	120.10 (18)
N1—P1—C15	110.52 (7)	C11—C12—H12	120.0
C14—P1—C15	108.36 (8)	C13—C12—H12	120.0
N1—P1—C8	111.53 (7)	C12—C13—C8	119.40 (16)
C14—P1—C8	109.37 (7)	C12—C13—H13	120.3
C15—P1—C8	109.55 (7)	C8—C13—H13	120.3
C6—N1—P1	125.47 (10)	P1—C14—H14A	109.5
C6—N1—H1N	116.5 (17)	P1—C14—H14B	109.5
P1—N1—H1N	117.9 (17)	H14A—C14—H14B	109.5
C2—C1—C6	119.74 (13)	P1—C14—H14C	109.5
C2—C1—S1	119.26 (11)	H14A—C14—H14C	109.5
C6—C1—S1	120.80 (11)	H14B—C14—H14C	109.5
C1—C2—C3	121.75 (14)	P1—C15—H15A	109.5
C1—C2—H2	119.1	P1—C15—H15B	109.5
C3—C2—H2	119.1	H15A—C15—H15B	109.5
C4—C3—C2	117.74 (14)	P1—C15—H15C	109.5
C4—C3—C7	121.16 (14)	H15A—C15—H15C	109.5
C2—C3—C7	121.05 (14)	H15B—C15—H15C	109.5
C5—C4—C3	121.29 (14)	C17—C16—C21	119.3 (2)
C5—C4—H4	119.4	C17—C16—H16	120.3
C3—C4—H4	119.4	C21—C16—H16	120.3
C4—C5—C6	120.79 (14)	C16—C17—C18	121.3 (2)
C4—C5—H5	119.6	C16—C17—H17	119.4
C6—C5—H5	119.6	C18—C17—H17	119.4
C5—C6—C1	118.64 (13)	C19—C18—C17	119.44 (19)
C5—C6—N1	120.69 (13)	C19—C18—H18	120.3
C1—C6—N1	120.67 (13)	C17—C18—H18	120.3
C3—C7—H7A	109.5	C20—C19—C18	120.70 (19)
C3—C7—H7B	109.5	C20—C19—H19	119.7
H7A—C7—H7B	109.5	C18—C19—H19	119.7
C3—C7—H7C	109.5	C19—C20—C21	120.49 (19)
H7A—C7—H7C	109.5	C19—C20—H20	119.8
H7B—C7—H7C	109.5	C21—C20—H20	119.8
C13—C8—C9	120.36 (15)	C16—C21—C20	118.63 (18)
C13—C8—P1	118.82 (12)	C16—C21—H21	120.7
C9—C8—P1	120.78 (12)	C20—C21—H21	120.7
			
O1—S1—O2—O2	0.00 (7)	C2—C1—C6—N1	−178.06 (13)
O3—S1—O2—O2	0.00 (4)	S1—C1—C6—N1	−3.16 (19)
C1—S1—O2—O2	0.00 (6)	P1—N1—C6—C5	−29.7 (2)
C14—P1—N1—C6	−160.95 (12)	P1—N1—C6—C1	149.70 (12)
C15—P1—N1—C6	−42.91 (15)	N1—P1—C8—C13	30.59 (14)
C8—P1—N1—C6	79.22 (14)	C14—P1—C8—C13	−88.10 (13)
O1—S1—C1—C2	96.24 (13)	C15—P1—C8—C13	153.27 (12)
O3—S1—C1—C2	−25.20 (13)	N1—P1—C8—C9	−151.94 (12)
O2—S1—C1—C2	−144.27 (12)	C14—P1—C8—C9	89.38 (14)
O2—S1—C1—C2	−144.27 (12)	C15—P1—C8—C9	−29.26 (15)
O1—S1—C1—C6	−78.68 (13)	C13—C8—C9—C10	−0.3 (2)
O3—S1—C1—C6	159.88 (12)	P1—C8—C9—C10	−177.74 (13)
O2—S1—C1—C6	40.81 (13)	C8—C9—C10—C11	−0.4 (3)
O2—S1—C1—C6	40.81 (13)	C9—C10—C11—C12	0.5 (3)
C6—C1—C2—C3	0.7 (2)	C10—C11—C12—C13	−0.1 (3)
S1—C1—C2—C3	−174.27 (11)	C11—C12—C13—C8	−0.6 (3)
C1—C2—C3—C4	−1.8 (2)	C9—C8—C13—C12	0.8 (2)
C1—C2—C3—C7	175.96 (14)	P1—C8—C13—C12	178.27 (13)
C2—C3—C4—C5	0.9 (2)	C21—C16—C17—C18	−3.6 (4)
C7—C3—C4—C5	−176.87 (15)	C16—C17—C18—C19	1.1 (3)
C3—C4—C5—C6	1.1 (2)	C17—C18—C19—C20	2.6 (3)
C4—C5—C6—C1	−2.2 (2)	C18—C19—C20—C21	−3.7 (3)
C4—C5—C6—N1	177.15 (14)	C17—C16—C21—C20	2.5 (3)
C2—C1—C6—C5	1.3 (2)	C19—C20—C21—C16	1.1 (3)
S1—C1—C6—C5	176.21 (11)		

### Hydrogen-bond geometry (Å, º)

**Table:**

*D*—H···*A*	*D*—H	H···*A*	*D*···*A*	*D*—H···*A*
N1—H1*N*···O2	0.80 (2)	2.09 (2)	2.7374 (17)	139 (2)
N1—H1*N*···O2^i^	0.80 (2)	2.47 (2)	3.0311 (17)	128 (2)

Symmetry code: (i) −*x*+1, −*y*+1, −*z*+1.
